# A deep-learning system to help make the surgical planning of coil embolization for unruptured intracranial aneurysms

**DOI:** 10.1186/s41016-023-00339-y

**Published:** 2023-09-11

**Authors:** Xin Nie, Yi Yang, Qingyuan Liu, Jun Wu, Jingang Chen, Xuesheng Ma, Weiqi Liu, Shuo Wang, Lei Chen, Hongwei He

**Affiliations:** 1 Department of Neurosurgery, Beijing Tiantan Hospital, Capital Medical University, Beijing, 100050 China; 2 China National Clinical Research Center for Neurological Diseases, Beijing, 100050 China; 3 Unimed Technology (Beijing) Co., Ltd., Tsinghua Tongfang Science and Technology Mansion, Beijing, 100083 China; 4 Department of Neurosurgery, The First Dongguan Affiliated Hospital, Guangdong Medical University, No. 42 Jiaoping Road, Tangxia Town, Dongguan, Guangdong China; 5 Beijing Neurosurgical Institution, Capital Medical University, Beijing, 100050 China

## Abstract

**Background:**

Coil embolization is a common method for treating unruptured intracranial aneurysms (UIAs). To effectively perform coil embolization for UIAs, clinicians must undergo extensive training with the assistance of senior physicians over an extended period. This study aimed to establish a deep-learning system for measuring the morphological features of UIAs and help the surgical planning of coil embolization for UIAs.

**Methods:**

Preoperative computational tomography angiography (CTA) data and surgical data from UIA patients receiving coil embolization in our medical institution were retrospectively reviewed. A convolutional neural network (CNN) model was trained on the preoperative CTA data, and the morphological features of UIAs were measured automatically using this CNN model. The intraclass correlation coefficient (ICC) was utilized to examine the similarity between the morphologies measured by the CNN model and those determined by experienced clinicians. A deep neural network model to determine the diameter of first coil was further established based on the CNN model within the derivation set (75% of all patients) using neural factorization machines (NFM) model and was validated using a validation set (25% of all patients). The general match ratio (the difference was within ± 1 mm) between the predicted diameter of first coil by model and that used in practical scenario was calculated.

**Results:**

One-hundred fifty-three UIA patients were enrolled in this study. The CNN model could diagnose UIAs with an accuracy of 0.97. The performance of this CNN model in measuring the morphological features of UIAs (i.e., size, height, neck diameter, dome diameter, and volume) was comparable to the accuracy of senior clinicians (all ICC > 0.85). The diameter of first coil predicted by the model established based on CNN model and the diameter of first coil used actually exhibited a high general match ratio (0.90) within the derivation set. Moreover, the model performed well in recommending the diameter of first coil within the validation set (general match ratio as 0.91).

**Conclusion:**

This study presents a deep-learning system which can help to improve surgical planning of coil embolization for UIAs.

**Supplementary Information:**

The online version contains supplementary material available at 10.1186/s41016-023-00339-y.

## Background

Intracranial aneurysm is the leading cause of nontraumatic subarachnoid hemorrhage [1, 2]. More than 70% of intracranial aneurysms are caused by unruptured intracranial aneurysms (UIAs) [3]. Coil embolization is among the most commonly used methods to treat UIAs [4, 5]. Nevertheless, due to the potential for incomplete embolization and coil displacement resulting in recurrent UIAs after embolization, neuro-interventionists require extensive clinical training with the guidance of experienced physicians over an extended period [6, 7].

The first coil plays an important role in determining the stability of intra-aneurysmatic embolization system [8–12]. Appropriate first coil can provide an enough space for subsequent coils and prevent embolization system from displacement [11]. How to choose appropriate first coils require accurate measurement of morphological features (e.g., aneurysm size, height, and dome diameter) and extensive clinical experience [9]. These limitations hinder clinicians from small or inexperienced medical centers to perform coil embolization for UIAs, and hence, some UIA patients do not benefit from this surgery.

In this preliminary study, we retrospectively reviewed the radiological features and surgical data of UIA patients undergoing coil embolization in our medical institution. Based on the preoperative computational tomography angiography (CTA) data and the first coils used in practical operations, we developed a deep-learning system for determining the morphological features of UIAs and recommend the appropriate first coils for UIA embolization.

## Methods

### Patient selection

**Fig. 1:**
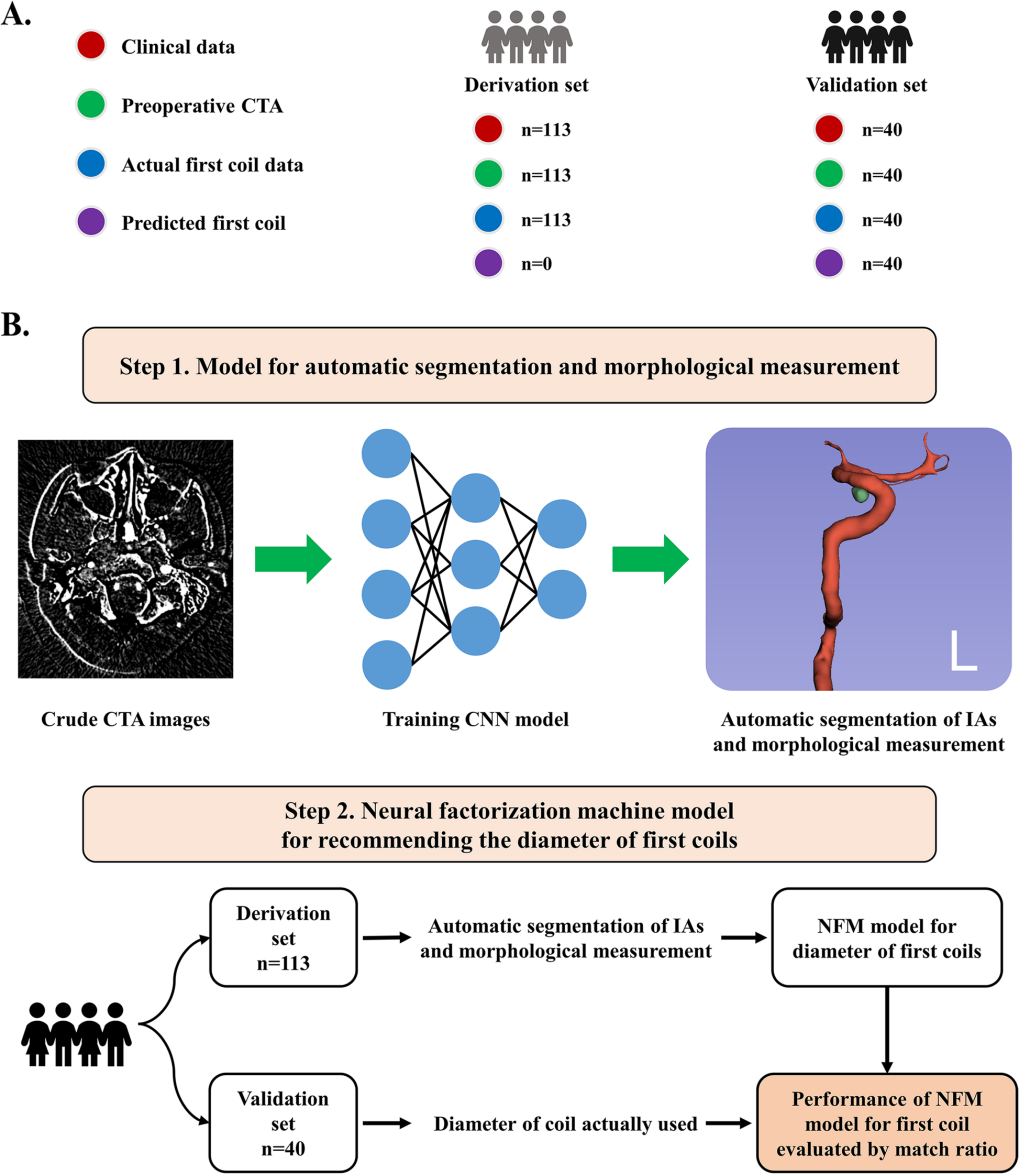
Summary of data generation and study design. **A** The summary of data generation. One-hundred thirteen UIA patients receiving neuro-interventional surgery were included as the derivation cohort, and 40 patients were included as the validation cohort. **B** Study design. In this study, we firstly established a CNN model to automatically measure the morphological features of UIAs using the U-Net algorithm. Subsequently, a NFM model was established for recommending the diameter of first coils. UIA, intracranial aneurysm; CNN, convolutional neural network; NFM, neural factorization machines

Patients undergoing coil embolization for UIAs in our medical institution from November 2022 to February 2023 were retrospectively enrolled. The inclusion criteria were as follows: (1) patients aged 18–80 years old, (2) unruptured aneurysms and without history of subarachnoid hemorrhage, (3) UIAs were treated by coil embolization or coil embolization assisted by stent, and (4) the medical record was complete or could be traced. Patients matching the following criteria were excluded: (1) irregular UIAs (bleb or secondary aneurysm protruding or bi-/multi-lobular aneurysm fundus) [13]; (2) tiny UIAs (< 3 mm) or large UIAs (> 10 mm); (3) combined cerebrovascular malformations (e.g., arteriovenous malformation) or intracranial tumors (e.g., meningioma and glioma); (4) significant stenosis in the parent artery of treated UIAs; (5) received UIAs treatment at the cavernous sinus segment of carotid artery; (6) UIAs were fusiform, dissecting, traumatic, bacterial, or atrium myxomas aneurysms; and (7) UIAs were treated by flow diverters. A quality screening for CTA images was conducted which resulted in a further exclusion of cases with (1) image quality assessment score < 3 [14] and (2) abnormal artifacts in images. The data processing and preparation procedure are presented in Fig. 1A. All patients were further classified as the derivation set (patients included from November 2022 to December 2022) and validation set (patients included from January 2023 to February 2023).

### Study design

This preliminary study aimed to establish a model for predicting morphological features and recommending the diameter of the first coils for embolization. The study design is presented in Fig. 1B. Based on images from all included patients, we constructed a model for the segmentation of UIAs and measurement of morphological features by using the convolutional neural network (CNN) model. The consistency between morphological features (including aneurysm location, size, height, volume, dome diameter and neck diameter) measured by senior doctors and those predicted by the model was compared. Subsequently, we established and validated a model for recommending the diameter of first coils using the neural factorization machines (NFM) model. The match ratio between diameter of coil actually used and coil predicted by model was also explored to verify the performance of NFM model.

### Data collection and measurement of morphological features

Demographic information such as age and gender, as well as the size of the initial coils employed, was obtained from the electronic medical records. All CTA source images were acquired using a CT scanner (Siemens Healthineers; Erlangen, Germany) under the following parameters: slice thickness of 0.625 mm, a field of view of 25 mm, 256 slices, resolution of 512 × 512, window center/window width as 400/40, tube voltage of 100–120 kV, and tube current of 500–600 mA. Scan time was < 5 s. Preoperative CTA data were collected as the digital imaging and presented in medical format. CTA data were reconstructed as the three-dimensional models using the Mimics 17.0 (Materialize, Belgium). Two neuro-interventionists (with > 15 years of work experience) measured the measured morphological features based on three-dimensional models and were blinded to the clinical information. Any discrepancies between the two investigators were solved by consulting a senior neuro-interventionist (with > 20 years of experience) who was also blinded to patients’ clinical information. The morphological features measured included aneurysm location, size, height, volume, dome diameter, and neck diameter. The location was categorized as anterior communicating artery (Acom)/anterior cerebral artery (ACA), internal carotid artery (ICA), middle cerebral artery (MCA), or posterior circulation (PC).

### Automatic segmentation

The UIA was manually segmented using the 3D Slicer (www.slicer.org). Two neuroradiologists delineated intracranial aneurysm slice by slice in the axial direction of subtract images (post-contrast minus pre-contrast) on the 3D Slicer. Aneurysms were delineated based on their texture of light and dark reflected by vessel (contrasted). A total of 153 UIAs were labeled and randomly assigned to the training images (75%, *n* = 113) and testing images (25%, *n* = 40).

The neural network is developed using the U-Net method to segmentate the UIAs in the subtract CTA [15]. The process of segmenting involved an end-to-end approach where the model was fed with subtracted CTA images as input, and produced masks of UIAs as output, which were of the same dimensions as the input images. During data preprocessing, we magnified the images to the size of 224 × 256 × 256 by using bilinear interpolation. To estimate the distribution of input images, data augmentation, including rotating, flipping, zooming, brightness and contrast adjusting, and elastic deformation, was conducted based on the training images. Subsequently, we combined residual connections for each convolution block. Max pooling operation was applied to each layer of the encoder part, and the transposed convolution was performed in the decoder part. Finally, a feature map with the size of 16 × 16 × 16 was obtained in the bottom layer. After each convolution, group normalization and ReLU activation function were applied. During the training process, cross-entropy combined with exponential logarithmic loss was conducted. Losses were optimized using the Adam method. The learning rate was initialized as 0.001, and the batch size was 14. The model was trained in 300 epochs with 400 steps in each epoch.

The model’s performance was evaluated based on the dice score, precision, F2 score, and recall score. True positive (TP), false positive (FP), and false negative (FN) were then calculated. The dice, precision, recall, and F2 were defined as follows:

$$\mathrm{Dice}=\frac{2\mathrm{TP}}{2\mathrm{TP}+\mathrm{FP}+\mathrm{FN}}$$

$$\mathrm{Precision}=\frac{\mathrm{TP}}{\mathrm{TP}+\mathrm{FP}}$$

$$\mathrm{Recall}=\frac{2\mathrm{TP}}{\mathrm{TP}+\mathrm{FN}}$$

$$\mathrm{F}2=\frac{5\times \mathrm{precision}\times \mathrm{recall}}{4\times (\mathrm{precision}+\mathrm{recall})}$$

The morphological features, including aneurysm size, height, neck and dome diameter, and volume, were measured using the result of segmentation of UIAs obtained by the CNN model. The aneurysm size was the gravity center of neck plane to the farthest point of aneurysm dome [16]. The aneurysm height was considered the maximum perpendicular distance of the dome from the neck plane [16]. The diameter of neck was taken as the average of lines passing through geometric center of neck plane, and the diameter of dome was the average of lines passing through geometric center of largest plane paralleling to the neck plane. The aneurysm volume was reflected by the volume of the segmented dome [16].

### The strategy to establish model for the diameter of first coils

A deep-learning (DL) model was established using NFM [17] in the Python (version 3.10). The prediction target was the diameter of the actual coils used. In total, 113 patients in the derivation set were randomly assigned into the training set (90 patients, 80%) and testing set (23 patients, 20%). The framework of each NFM node is provided in Supplemental Fig. 4. The NFM model was trained based on selected features through a fivefold cross-validation. To further assess overfitting, a learning curve of the NFM model was developed based on the percentage of general matched cases (the difference between the diameter of coils predicted and the diameter of coils actually used was within ± 1 mm) of the training and testing set. An average of training percentages of all repetitions (based on the training set) was applied, and 95% confidence interval (CI) was calculated based on the results of the fivefold cross-validation. If the testing percentage, determined from the testing set, fell within the 95% confidence interval of the training percentage, then it was concluded that the model was not overfitting. The performance of model in recommending the diameter of first coils was validated externally using a different validation set.

The absolute match ratio and general match ratio were used to evaluate the performance of NFM model in recommending the diameter of first coils. The absolute matched cases were identified when the diameter of coils predicted perfectly matched that of the actual coils used. Additionally, cases where the predicted coil diameter differed from the actual diameter by no more than ± 1 mm were categorized as matches. The absolute and general match ratios were defined as follows:

$$\mathrm{Absolute\,match\,ratio}=\frac{\mathrm{number\,of\,absolute\,matched\,cases\,}(\mathrm{perfectly\,matched})}{\mathrm{all\,patients}}$$

$$\mathrm{General\,match\,ratio}=\frac{\mathrm{number\,of\,general\,matched\,cases\,}(\mathrm{within}\pm 1\mathrm{\,mm})}{\mathrm{all\,patients}}$$

### Statistics analysis

Statistical analyses were conducted with SPSS (version 24.0, Chicago, USA). Continuous data with normal distribution were analyzed using the Shapiro–Wilk test. Continuous variables with normal distribution were presented as means and standard deviation. Data that did not follow normal distribution were presented as the medians and interquartile range (IQR). Categorical variables were presented as numbers (n) and percentage (%). Differences between continuous variables were compared by using Student’s *t*-tests or Wilcoxon rank-sum tests, and differences among categorical variables were analyzed using chi-square tests or Fisher’s exact tests. The reproducibility (between two investigators) and consistency (between investigators and model) of morphological features (aneurysm size, height, neck and dome diameter, and volume) were evaluated using intraclass correlation coefficient (ICC). In these analyses, *ICC* > 0.8 was considered a good consistency.

## Results

### Baseline information of all included patients.

A total of 153 patients were enrolled from 236 UIA patients undergoing coil embolization (Supplemental Fig. 1). The clinical and radiological information of all patients are presented in Table 1. The median age of patients was 58 (range, 35–85) years, and 39 (25.5%) patients were male. Among all UIAs, the median aneurysm size was 5.1 (3.0–9.6) mm and 47 (30.7%) UIAs sited in Acom/ACA, 73 (47.7%) in ICA, 21 (13.7%) in MCA, and 12 (7.8%) in PC. The reproducibility of the measurement of morphological features between two investigators is shown in Supplemental Table 1.

**Table 1:** Baseline information of all included patients

Characteristics	All patients *n* = 153	Derivation set *n* = 113	Validation set *n* = 40
Age, median (IQR), years	58 (66–52)	58 (67–52)	59 (66–50)
Male, *n* (%)	39 (25.5%)	26 (23.0%)	13 (32.5%)
UIA location, *n* (%)			
Acom/ACA	47 (30.7%)	37 (32.7%)	10 (25.0%)
ICA	73 (47.7%)	54 (47.8%)	19 (47.5%)
MCA	21 (13.7%)	13 (11.5%)	8 (20.0%)
PC	12 (7.8%)	9 (8.0%)	3 (7.5%)
UIA size, median (IQR), mm	5.1 (6.6–3.6)	4.2 (6.2–3.4)	6.4 (6.7–5.9)
UIA height, median (IQR), mm	3.0 (4.0–2.3)	3.3 (4.1–2.4)	2.5 (3.5–2.2)
Neck diameter, median (IQR), mm	2.0 (2.6–1.0)	1.5 (2.2–0.9)	2.7 (3.8–2.4)
Dome diameter, median (IQR), mm	6.4 (8.2–3.2)	4.8 (6.9–2.7)	8.3 (11.8–7.5)
UIA volume, median (IQR), mm	44.9 (18.6–128.1)	27.9 (13.5–53.6)	48.7 (29.9–110.3)

*UIA* Unruptured intracranial aneurysm, *Acom* Anterior communicating artery, *ACA* Anterior cerebral artery, *ICA* Internal carotid artery, *MCA* Middle cerebral artery, *PC* Posterior circulation

### An automatic model for morphological measurement of UIAs

**Fig. 2:**
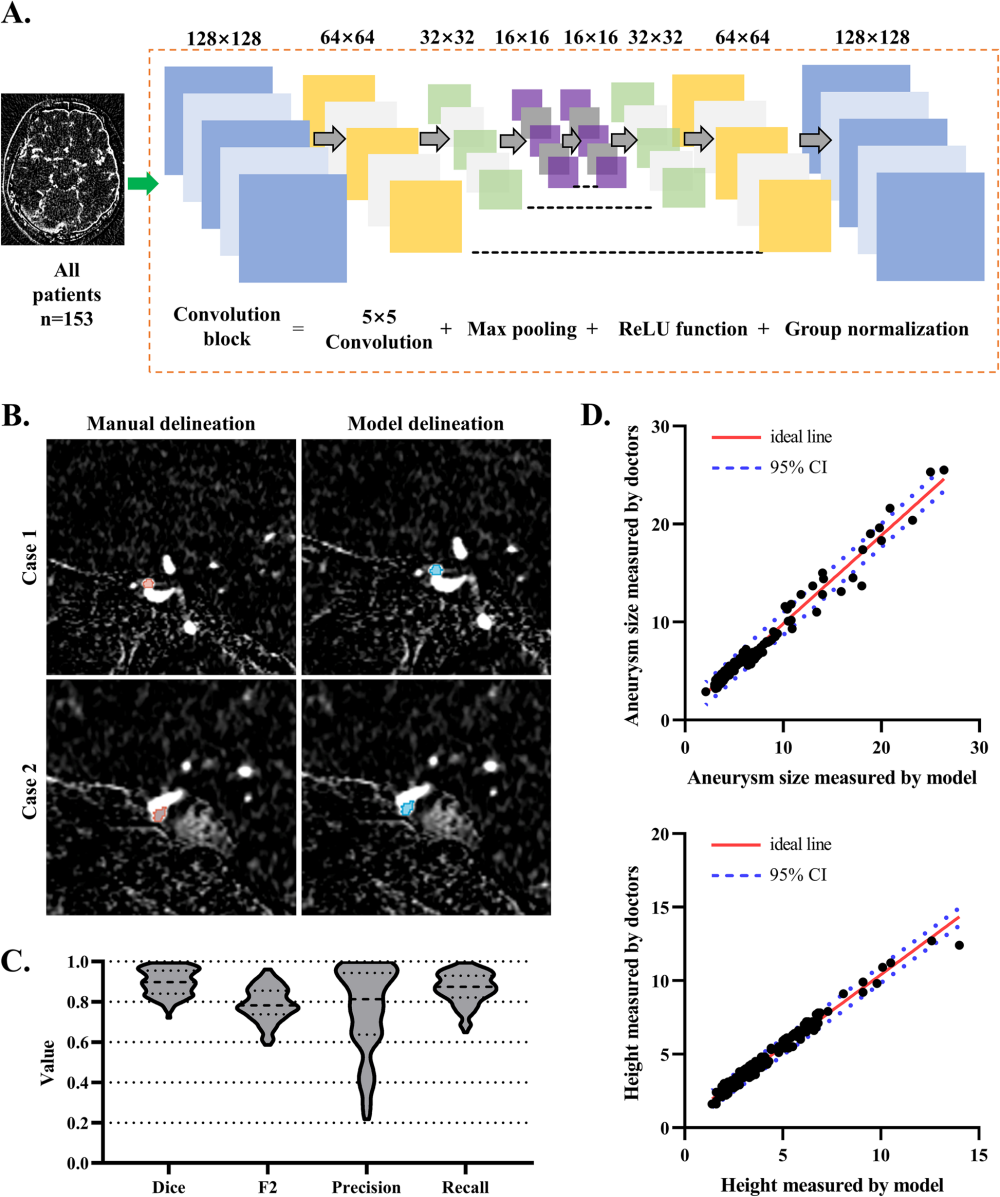
Establishment of an automatic morphological measurement model. **A** The flowchart of establishing CNN model to identify and segmentate UIAs. **B** The manual and model delineation images of representative cases. **C** The violin plots present the distribution of dice, F2, precision, and recall value within the testing set (*n* = 40). **D** The scatter dot plots present the correlation of aneurysm size and height measured by model and doctors. UIA, unruptured intracranial aneurysm; CNN, convolutional neural network

The CNN model for measuring morphological features of UIAs was trained (our U-Net-based segmentation model on the images from 153 included patients as shown in Fig. 2A). CTA-sourced images were randomly divided into the training images (75%, *n* = 113) and testing images (25%, *n* = 40). The baseline information is shown in Table 1. The images of manual delineation and model delineation of two representative cases are illustrated in Fig. 2B. The dice between manual delineation and model delineation was 0.90 within the testing images (Fig. 2C), suggesting that the CNN model performed well in identifying UIAs. Further analysis showed that the accuracy of model to diagnosis UIAs was 0.94 within the testing images and was 0.97 for images from all patients (Supplemental Fig. 2A).

Using the three-dimensional model constructed by the CNN model, morphological features of UIAs (i.e., size, height, neck diameter, dome diameter, and volume) (the diagram of morphological measurement after UIA segmentation was given in Supplemental Fig. 2B) were measured. We obtained a good consistency (ICC value more than 0.85) between the morphological features measured by model and by doctors, within the training images and testing images (Fig. 2D, also see Supplemental Fig. 2C).

### A deep-learning model for the diameter of first coils

**Fig. 3:**
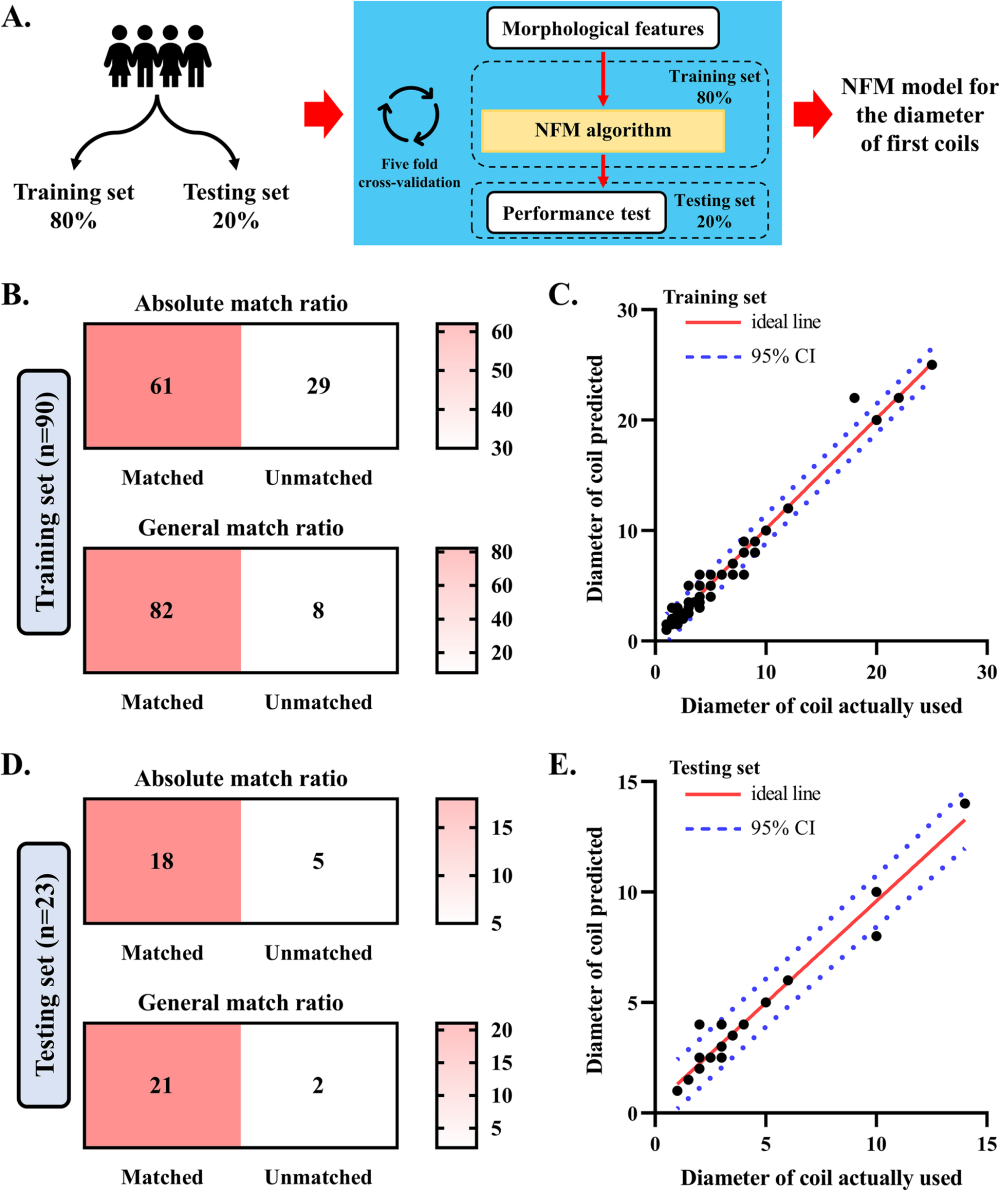
Establishment of a DL model for the diameter of first coils. **A** The flowchart of establishing NFM model for the diameter of first coils. Patients in the derivation set were grouped as the training set (80%, *n* = 90) and testing set (20%, *n* = 23). Based on the training set, a model for the diameter of first coils was trained. The accuracy of this model was validated based on the testing set. **B** The performance of model for recommending the diameter of first coils within the training set. The absolute match ratio was 67.8% (61/90), and the general match ratio was 91.1% (82/90). **C** The scatter dot plots present the correlation of diameter of coil actually used and coil predicted by model within the training set. **D** The performance of model for recommending the diameter of first coils within the training set. The absolute match ratio was 78.2% (18/23), and the general match ratio was 91.3% (21/23). **E** The scatter dot plots present the correlation of diameter of coil actually used and coil predicted by model within the testing set. NFM, neural factorization machines

A deep-learning model for recommending the diameter of first coils using the NFM was further constructed using the morphological features of UIAs measured by the CNN model (Fig. 3A). All patients were grouped either to the derivation set (75% of all patients) and validation set (25% of all patients) (the baseline information was given in Table 1). The model was trained on the derivation set. The match ratio between the diameter of actual coils used and predicted by NFM model was investigated, and the results are presented in Table 2. For the training set, the absolute match ratio (perfect match) was 0.67 (61/90), and the general match ratio (within ± 1 mm) was 0.91 (82/90) (Fig. 3B). A good consistency was obtained between the diameter of actual coils used and those predicted by the NFM model (Fig. 3C). Within the testing set, the absolute match ratio was 0.78, and the general match ratio was 0.91 (Fig. 3D–E). Further analysis based on the learning curve revealed no overfitting issue in this NFM model (Supplemental Fig. 3).

**Table 2:** The performance of NFM model for the diameter of first coils

Data sets	Derivation cohort		Validation cohort
	Training set	Testing set	
Absolute match ratio	0.68	0.78	0.65
General match ratio	0.91	0.91	0.90

*NFM* Neural factorization machines

**Fig. 4:**
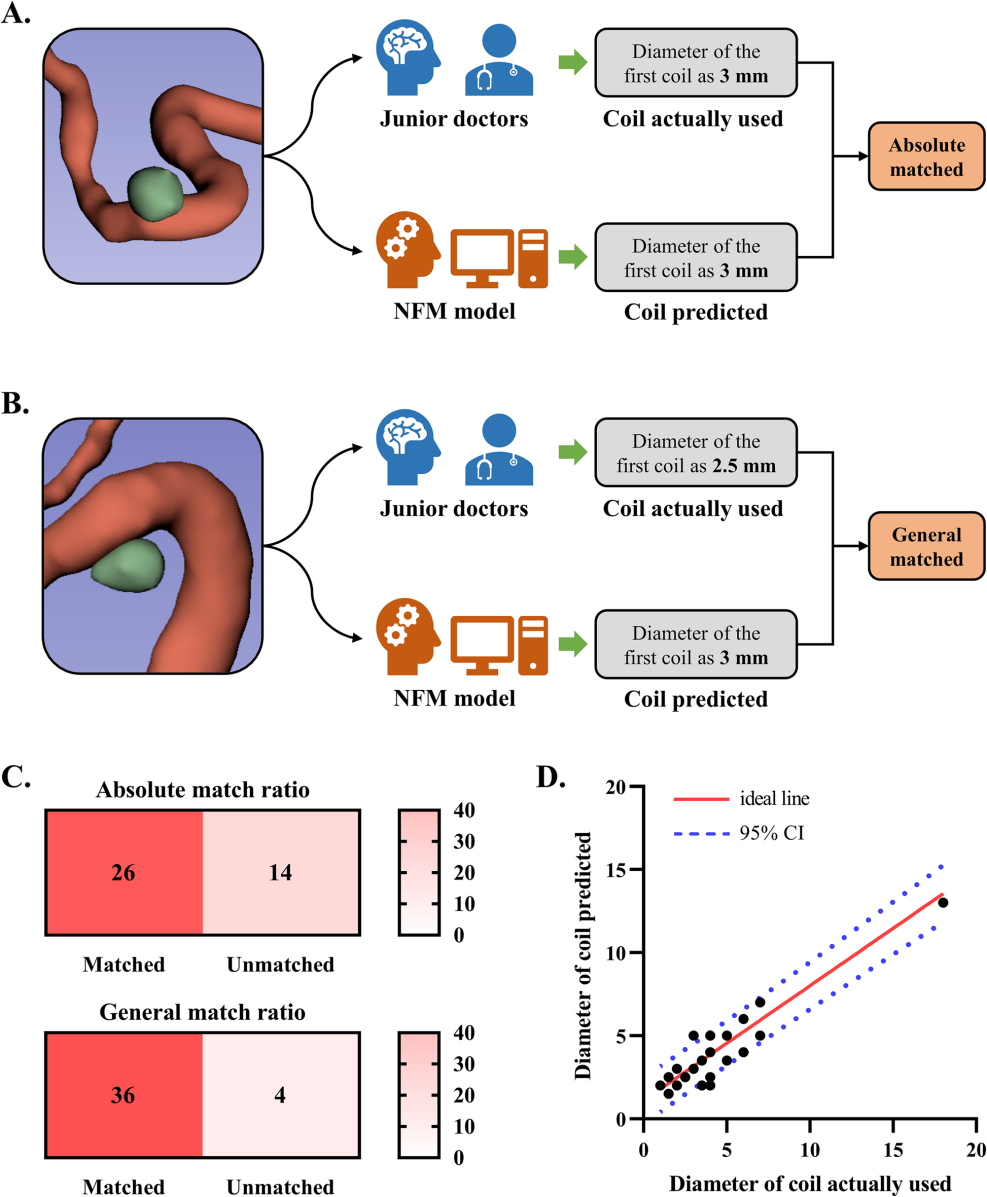
Validation of the NFM model for the diameter of first coils. **A** A representative case, whose diameter of first coil by model was absolutely matched the diameter of first coil actually used. **B** A representative case, whose diameter of first coil by model was generally matched the diameter of first coil actually used. **C** The performance of model for recommending the diameter of first coils within the validation set. The absolute match ratio was 65.0% (26/40), and the general match ratio was 90.0% (36/40). **D** The scatter dot plots present the correlation of diameter of coil actually used and coil predicted by model within the validation set. NFM, neural factorization machines

The accuracy of NFM model was validated in the validation set, and the evaluation process of representative cases is presented in Fig. 4A–B. For the validation set, the absolute match ratio was 0.65 (26/40), and the general match ratio was 0.90 (36/40) (Fig. 4C). A good consistency was obtained between the diameter of actual coils used and predicted by DL model for the validation cohort (Fig. 4D).

## Discussion

Using a suitable tool could reduce the amount of time it takes for clinicians to become proficient in coil embolization for UIAs and enhance the effectiveness of the embolization procedure. In this current study, we established a deep-learning system, including a CNN model and a NFM model. The CNN model had a high accuracy in identifying UIAs (accuracy as 0.97). Subsequent analysis showed that the morphological features measured by CNN model had a good consistency with the morphological features measured by senior neuro-interventionists (ICC value > 0.80). The NFM model established using the CNN model performed well in recommending the diameter of first coils, with general match ratio (within ± 1 mm) > 0.90. Thus, our deep-learning system may be clinically useful in guiding the surgical planning of coil embolization for UIAs.

Accurate measurement of morphological features of UIAs is essential to the surgical planning of coil embolization. Previous studies mainly focused on establishing models for diagnosing intracranial aneurysms [18–21]. While the previous deep-learning model demonstrated a diagnosis accuracy of > 0.9 for intracranial aneurysms, it also greatly enhanced the ability of neuroradiologists to diagnose such aneurysms [21]. However, few of these studies have explored whether the segmentation of intracranial aneurysms using their models can accurately measure morphological features. In this study, we first asked three experienced neuro-interventionists to measure morphological features as the “gold standard.” Subsequent analysis demonstrated a good consistency between the morphological features measured by our CNN model and those obtained by the senior clinicians. Unlike previous works, this study evaluated the performance of model in measuring the morphological features of UIAs. Thus, our CNN model can provide a reliable reference for the clinical measurement of morphological features.

Neuro-interventionists who can perform coil embolization for UIAs are required to undergo a lengthy clinical training [22]. Because small and inexperienced centers usually lack neuro-interventionists with experience in coil embolization for UIAs and surgical operation, we trained a NFM model based on the CNN model to recommend the diameter of first coils for UIA embolization. Results showed that the diameter of first coils recommended by our NFM model was generally matched with the diameter of first actual coils used. This demonstrated that our NFM model can help surgical planning of coil embolization for UIAs. By using this model, junior and inexperienced clinicians could learn how to measure the morphological features of UIAs accurately, as the same as guide the experienced clinicians. Subsequently, these medical professionals could engage with the NFM algorithm to determine the appropriate size of coils to use for the embolization of UIAs. Thus, our deep-learning system (including CNN model and NFM model) is likely to help trainers to deepen the understanding of morphological measurement of UIAs and identify ways for choosing appropriate first coil for UIA embolization. This may help shorten the training period for junior and inexperienced clinicians in learning about coil embolization for UIAs, despite the facts that small and inexperienced centers lack experienced neuro-interventionists.

In this study, CTA data were used to establish deep-learning model. Although not good as digital subtract angiography, CTA showed the morphological features of UIAs noninvasively compared with magnetic resonance angiography. In addition, it was observed that the NFM model based on the CTA source data performed well in predicting the diameter of first coil for UIA embolization. Thus, CTA source data may serve as a reliable reference to guide surgical planning preoperatively. However, further studies were needed to compare the accuracy of model based on CTA source data and model based on other data in measuring the morphological features of UIAs.

Although we present interesting findings, there are several limitations to this study. First, this work was based on a small sample and single-center study. Patient selection bias may also limit the quality of conclusions. Moreover, given that the parameters of CTA examination may be vary from center to center, thus the model established based on the data from single center may not be generalized to other centers. Second, our CNN model measured few morphological features of UIAs. There may be other morphological features related to the selection of the diameter of first coils. Third, this study did not evaluate the performance of NFM system on actual clinical condition. Whether the developed NFM system can improve the performance of junior and inexperienced clinicians in conducting coil embolization for UIAs needed to be further studied. Fourth, we excluded patients with large and irregular aneurysms, which may limit the generality of our conclusion.

## Conclusion

In this current study, we established a deep-learning system, which could measure the morphological features of UIAs and help make surgical planning of coil embolization for UIAs.

### Supplementary Information

**Additional file 1: Supplemental Fig. 1.** The flowchart of patient enrollment. Thins study finally enrolled 153 patients from 236 UIA patients. All included patients were grouped as the derivation cohort (113 UIA patients included from November 2022 to December 2022) and validation cohort (40 UIA patients included from January 2023 to February 2023). UIA, unruptured intracranial aneurysms. **Supplemental Fig. 2.** Establishment of an automatic morphological measurement model. A. The accuracy of model to diagnose and segmentate UIAs. Within the training set and testing set, the accuracy of model to diagnose UIAs was >0.90. B. The diagram of morphological measurement after UIA segmentation. Aneurysm size, height, dome diameter and neck diameter were measured by doctors and model. C. The consensus analysis of morphological measurement between doctors and model using the intraclass correlation coefficient method. UIA, unruptured intracranial aneurysm. **Supplemental Fig. 3.** The learning curve of NFM model for the diameter of first coil. **Supplemental Fig. 4.** The framework of each deep Neural Factorization Machines node. The morphological features of UIAs measured by the CNN model and coil features information as input, the embedding layer projects each feature to a dense vector representation. The upper layer are factorization machine layer and hidden layer which are capable of learning higher order interactions between features. At last, the results from factorization machine layer and hidden layer are integrated into intermediate node. UIA, unruptured intracranial aneurysm; CNN, convolutional neural network. **Supplemental Table 1.** The reproducibility of the measurement of morphological features between two investigators.

## Data Availability

The data supporting the findings of this study are available from the corresponding authors upon reasonable request.

## Abbreviations

UIA: Unruptured intracranial aneurysm
CTA: Computational tomography angiography
CNN: Convolutional neural network
DL: Deep learning
NFM: Neural factorization machines
Acom: Anterior communicating artery
ACA: Anterior cerebral artery
ICA: Internal carotid artery
ICC: Intraclass correlation coefficient
MCA: Middle cerebral artery
PC: Posterior circulation
CI: Confidence interval
