# Supplementary material for: A deep-learning system to help make the surgical planning of coil embolization for unruptured intracranial aneurysms
Source: Chin Neurosurg J. 2023 Sep 11;9:24. doi: 10.1186/s41016-023-00339-y (PMC10494453; doi:10.1186/s41016-023-00339-y)
Supplement: Supplementary file 1 — Additional file 1: Supplemental Fig. 1. The flowchart of patient enrollment. Thins study finally enrolled 153 patients from 236 UIA patients. All included patients were grouped as the derivation cohort (113 UIA patients included from November 2022 to December 2022) and validation cohort (40 UIA patients included from January 2023 to February 2023). UIA, unruptured intracranial aneurysms. Supplemental Fig. 2. Establishment of an automatic morphological measurement model. A. The accuracy of model to diagnose and segmentate UIAs. Within the training set and testing set, the accuracy of model to diagnose UIAs was >0.90. B. The diagram of morphological measurement after UIA segmentation. Aneurysm size, height, dome diameter and neck diameter were measured by doctors and model. C. The consensus analysis of morphological measurement between doctors and model using the intraclass correlation coefficient method. UIA, unruptured intracranial aneurysm. Supplemental Fig. 3. The learning curve of NFM model for the diameter of first coil. Supplemental Fig. 4. The framework of each deep Neural Factorization Machines node. The morphological features of UIAs measured by the CNN model and coil features information as input, the embedding layer projects each feature to a dense vector representation. The upper layer are factorization machine layer and hidden layer which are capable of learning higher order interactions between features. At last, the results from factorization machine layer and hidden layer are integrated into intermediate node. UIA, unruptured intracranial aneurysm; CNN, convolutional neural network. Supplemental Table 1. The reproducibility of the measurement of morphological features between two investigators. [file 41016_2023_339_MOESM1_ESM.docx]

Supplemental materials for “A deep-learning system to help make surgical planning of coil embolization for unruptured intracranial aneurysms”

**Supplemental tables**

**Supplemental table 1. The reproducibility of the measurement of morphological features between two investigators**

| Characteristics | Investigator 1 | Investigator 2 | ICC value  (95% CI) |
| --- | --- | --- | --- |
| UIA size, median (IQR), mm | 5.6 (4.2-7.4) | 5.7 (4.2-7.3) | 0.995 (0.994-0.997) |
| UIA height, median (IQR), mm | 4.4 (3.2-6.3) | 4.4 (3.1-6.4) | 0.983 (0.976-0.987) |
| Neck diameter, median (IQR), mm | 2.5 (1.6-3.0) | 2.5 (1.6-3.2) | 0.986 (0.981-0.990) |
| Dome diameter, median (IQR), mm | 6.9 (3.9-8.7) | 7.0 (3.8-8.6) | 0.998 (0.998-0.999) |
| UIA volume, median (IQR), mm | 0.7 (0.3-1.3) | 0.7 (0.3-1.2) | 0.970 (0.951-0.982) |

Abbreviation: UIA, unruptured intracranial aneurysm.

**Supplemental figures**


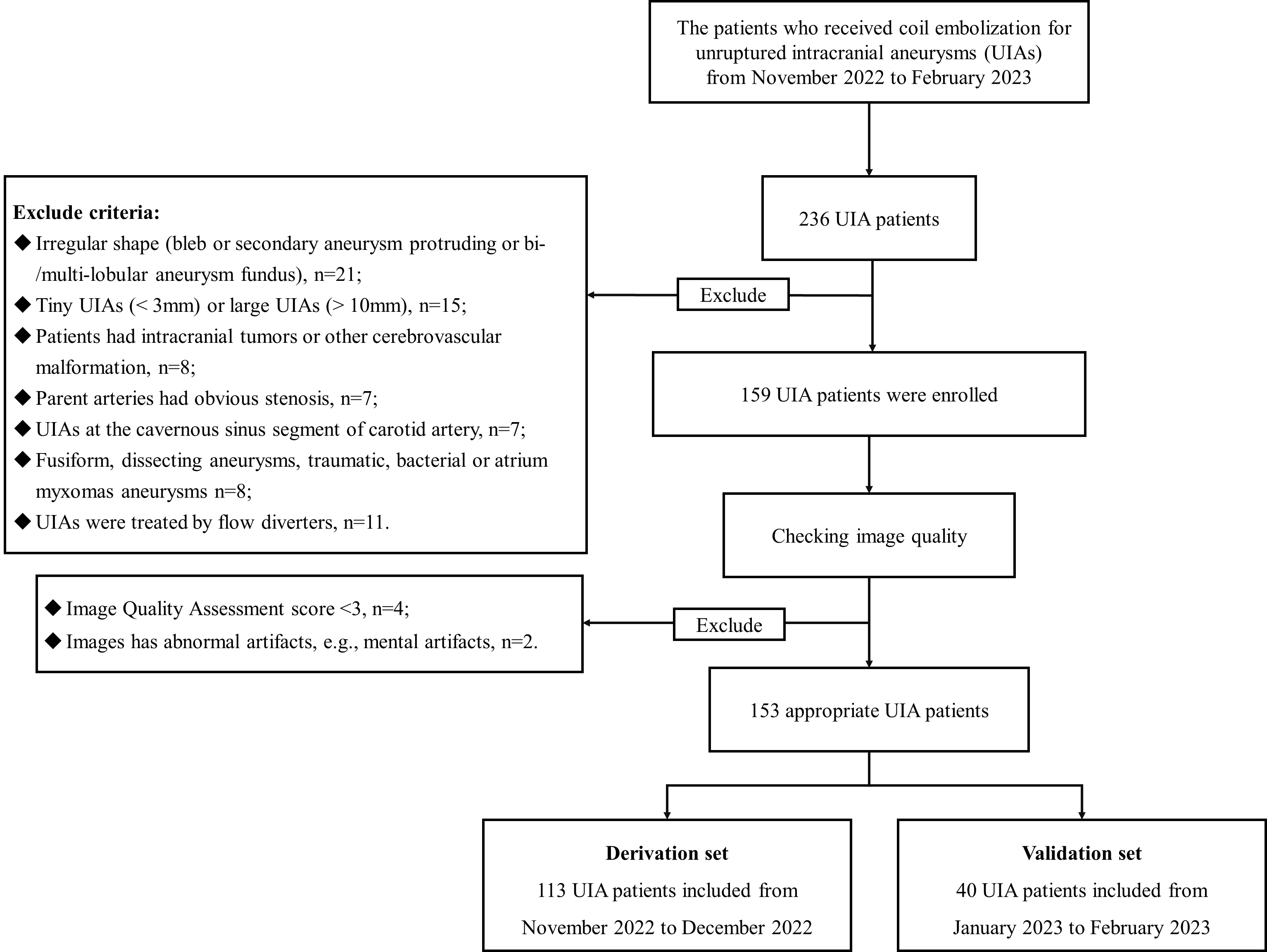


**Supplemental figure 1. The flowchart of patient enrollment.**

Thins study finally enrolled 153 patients from 236 UIA patients. All included patients were grouped as the derivation cohort (113 UIA patients included from November 2022 to December 2022) and validation cohort (40 UIA patients included from January 2023 to February 2023).

UIA, unruptured intracranial aneurysms.


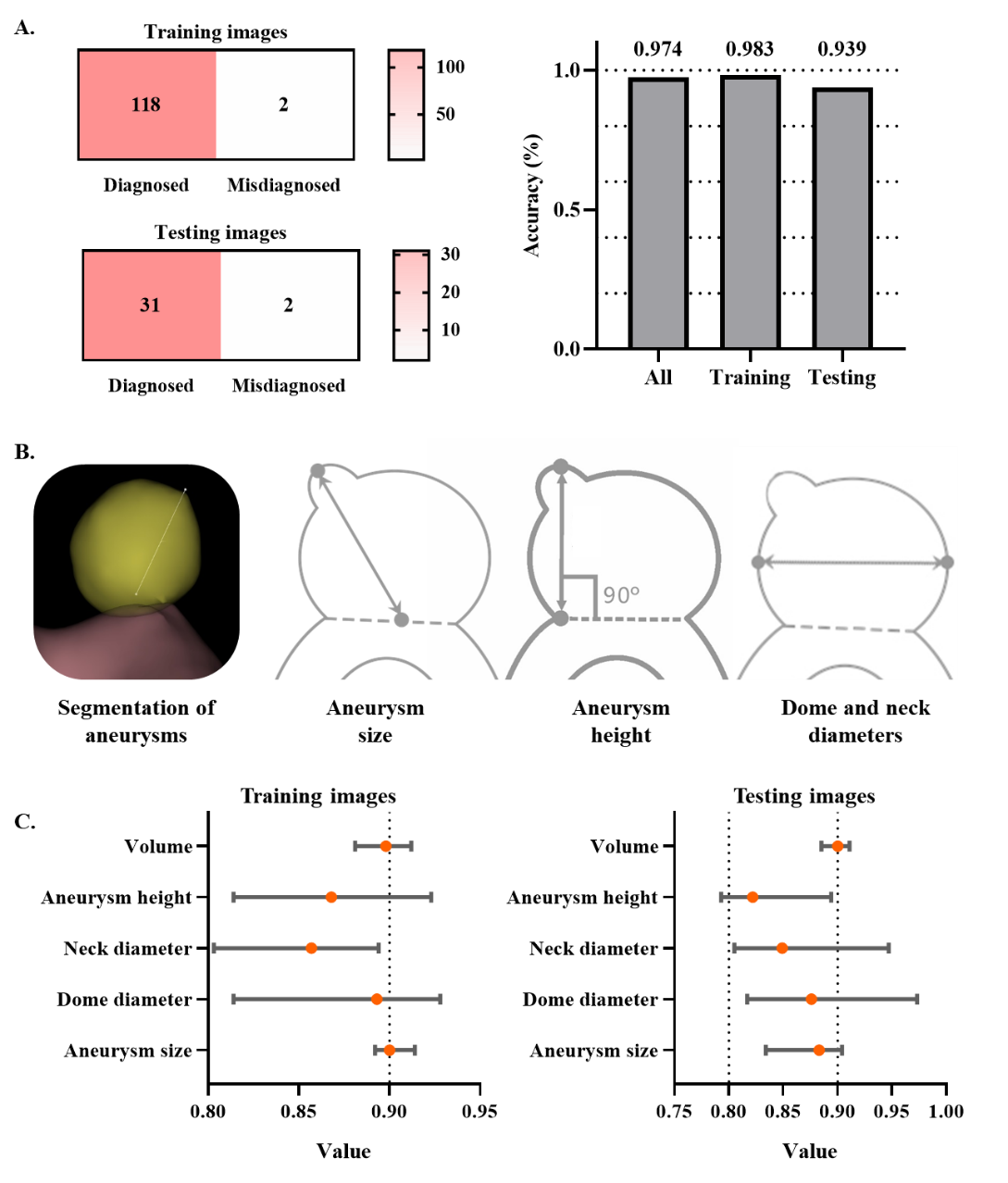


**Supplemental figure 2. Establishment of an automatic morphological measurement model.**

A. The accuracy of model to diagnose and segmentate UIAs. Within the training set and testing set, the accuracy of model to diagnose UIAs was >0.90.

B. The diagram of morphological measurement after UIA segmentation. Aneurysm size, height, dome diameter and neck diameter were measured by doctors and model.

C. The consensus analysis of morphological measurement between doctors and model using the intraclass correlation coefficient method.

UIA, unruptured intracranial aneurysm.


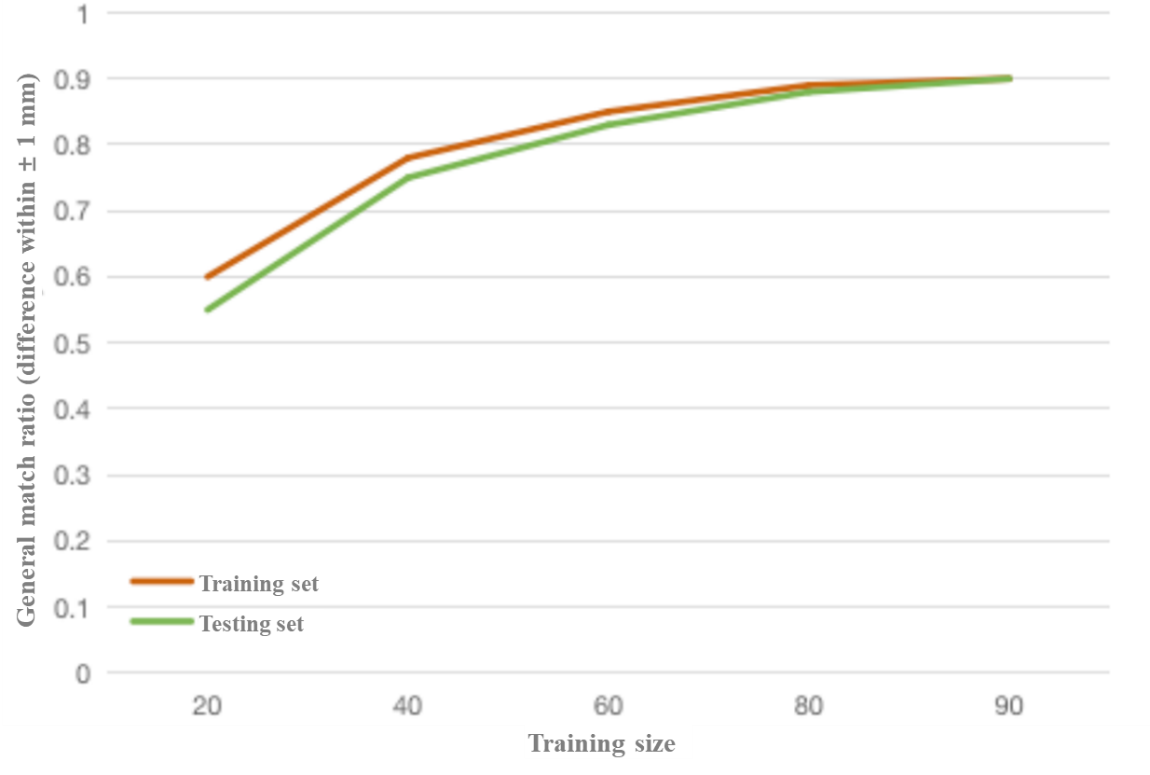


**Supplemental figure 3. The learning curve of NFM model for the diameter of first coil.**


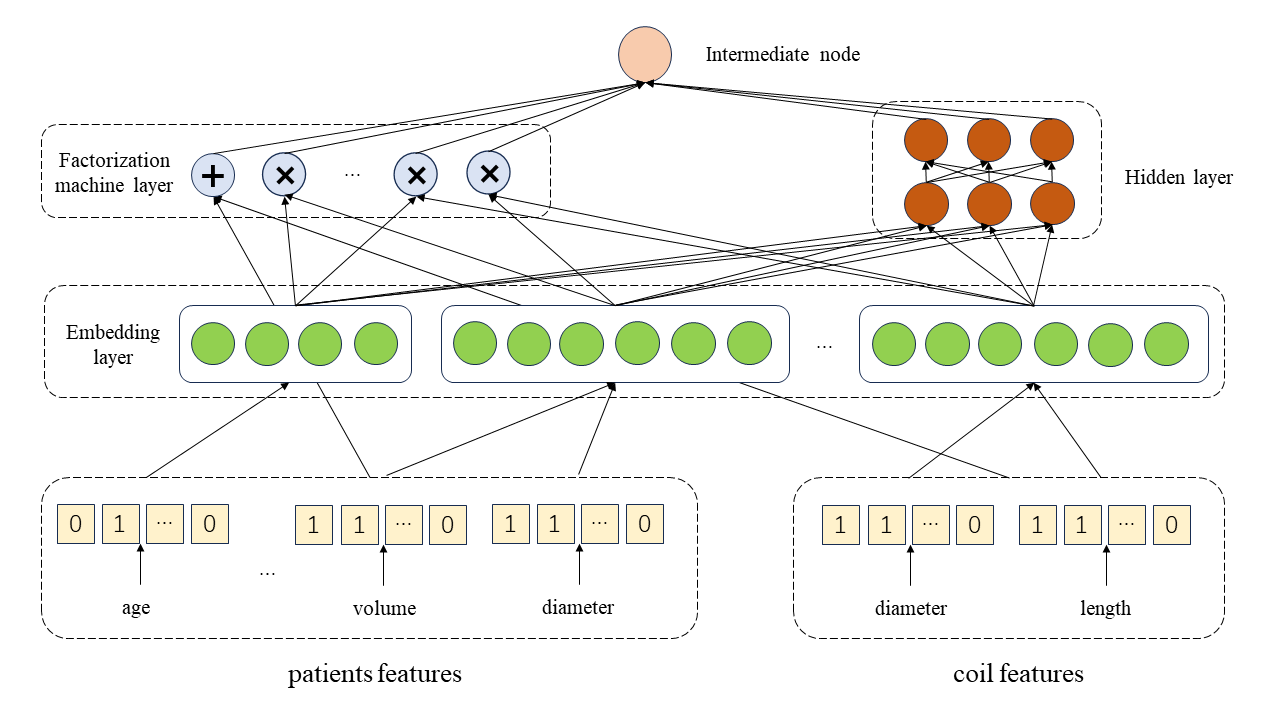


**Supplemental figure 4. The framework of each deep Neural Factorization Machines node.**

The morphological features of UIAs measured by the CNN model and coil features information as input, the embedding layer projects each feature to a dense vector representation. The upper layer are factorization machine layer and hidden layer which are capable of learning higher order interactions between features. At last, the results from factorization machine layer and hidden layer are integrated into intermediate node.

UIA, unruptured intracranial aneurysm; CNN, convolutional neural network.
